# Clinical outcome of different skin closure in total-knee arthroplasty: running subcuticular closure vs intermittent closure: A retrospective study

**DOI:** 10.1097/MD.0000000000021947

**Published:** 2020-08-21

**Authors:** Liang Chen, Junxiao Yang, Jie Xie, Yihe Hu, Min Zeng

**Affiliations:** Department of Orthopedics, Xiangya Hospital Central South University, Changsha, Hunan, China.

**Keywords:** clinical outcome, surgical-site infections, total-knee arthroplasty

## Abstract

An intermittent closure with silk suture is routinely used for closing different surgical wounds. However, subcuticular closure with absorbable sutures has gained considerable attention due to convenience and better cosmetic appearance.

To compare the clinical outcomes and risk of surgical-site infection of subcuticular and intermittent closure after total-knee arthroplasty (TKA), 106 patients that underwent TKA between January 2017 to June 2019 at the Department of Orthopedics in Xiangya Hospital of Centre South University were retrospectively assessed. Forty-three had received running subcuticular closure (group A) and 58 underwent intermittent closure (group B). The Knee Society score was measured before and 6 months after operation. Inflammation markers including the serum levels of procalcitonin, interleukin-6, and C-reactive protein, and the erythrocyte sedimentation rate were evaluated before operation, 1 day after and 1 month after operation. Patient satisfaction with the closure was evaluated using the Likert scale at the last follow-up.

No significant difference was seen in the 6-month postoperative Knee Society score, or in the 1-day and 6-month postoperative inflammation marker levels between both groups (*P* > .05). Likert scores were higher in group A compared to group B (4.0 ± 1.0 vs 3.6 ± 1.2, *P* < .05).

Running subcuticular closure after TKA results in a better appearance compared to intermittent closure, although neither method has an advantage in terms of efficacy and risk of infection.

## 1 Introduction

Poor skin closure after total-knee arthroplasty (TKA) may lead to pain, impaired physical activity and joint stiffness,^[1,2]^ in addition to surgical-site infections (SSIs) that may progress to a periprosthetic joint infection and bring economic burdens.^[3,4]^ In addition to running subcuticular or intermittent closure using staples or barbed sutures,^[5–7]^ a novel “zip” device and skin adhesives have also been explored for post-TKA skin closure.^[8,9]^ Although intermittent closure with silk suture is routinely used for different surgical wounds, subcuticular closure with absorbable sutures are increasingly being considered owing to their convenience and better cosmetic appearance. In this retrospective study, we compared the 6-month clinical outcome of running subcuticular closure and intermittent closure post-TKA.

## 2 Materials and methods

### 2.1 Patients

A total of 106 patients aged 50 to 81 years who had undergone primary TKA at the department of orthopedics in Xiangya Hospital of Centre South University (Reviewer #1 point 1) from January 2017 to June 2019 were enrolled after ethics committee review approval. All surgeries were performed by 1 surgeon. Patients that received bilateral TKA and those without knee osteoarthritis were excluded. Absorbable suture was used for running subcuticular closure in all surgeries until April 2018 (group A), whereas intermittent closure was performed using silk suture (group B). The final analysis was conducted on 101 patients since 5 were lost during the 6-month follow-up.

### 2.2 Surgical technique

**Figure 1:**
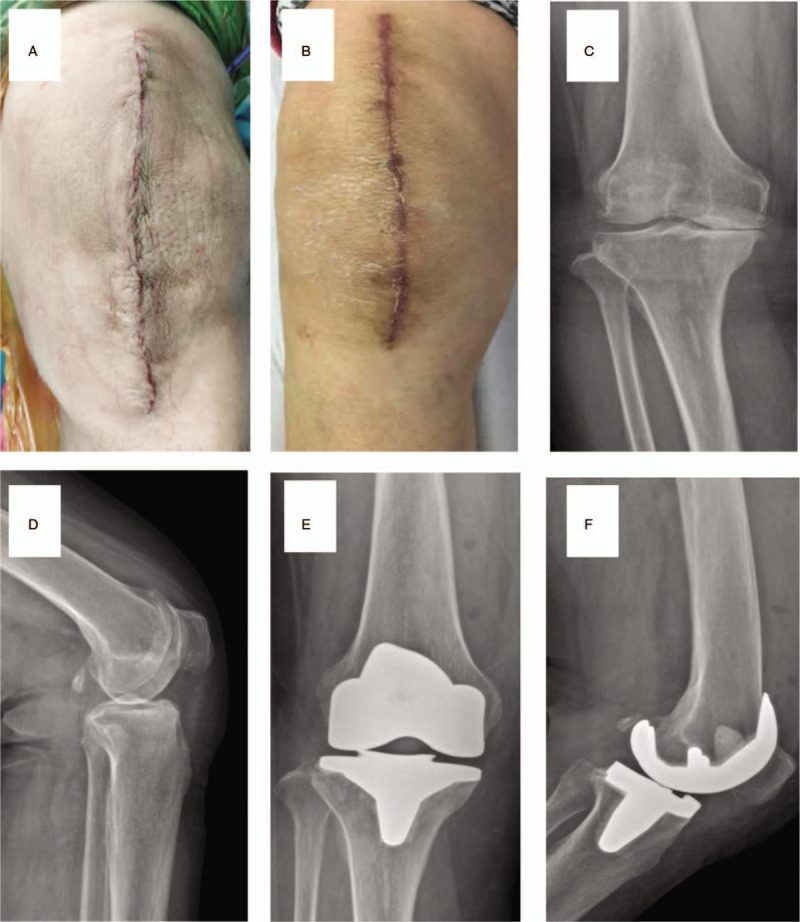
(A) Running subcuticular postoperation. (B) Running subcuticular closure at the last follow-up. (C) AP X-ray for knee in running subcuticular closure group preoperation. (D) Lateral X-ray for knee in running subcuticular closure group preoperation. (E) Lateral X-ray for knee in running subcuticular closure group at the last follow-up. (F) AP X-ray for knee in running subcuticular closure group at the last follow-up.

**Figure 2:**
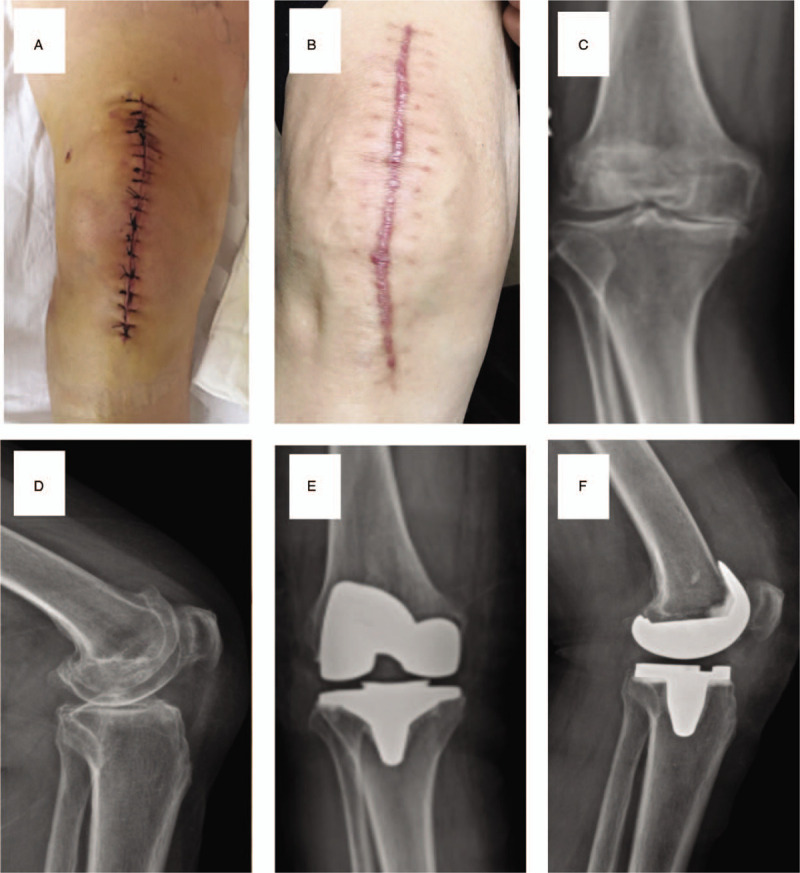
(A) Intermittent closure postoperation. (B) Intermittent closure at the last follow-up. (C) AP X-ray for knee in intermittent closure group preoperation. (D) Lateral X-ray for knee in intermittent closure group preoperation. (E) Lateral X-ray for knee in intermittent closure group at the last follow-up. (F) AP X-ray for knee in intermittent closure group at the last follow-up.

Cemented prostheses were implanted after osteotomy and a test model using the medial patellar approach. The length of incision ranged between 15 and 20 cm. After placing a drainage tube, the tendon, subcutaneous, and skin layers were sequentially closed with 0 absorbable line (VICRYL Plus VCP358 and Stratafix SXPP1A401), a 2-0 PDO (QUILL RA-1028Q), and 3-0 monoderm (YA-1023Q; group A) or silk (MERSILK W570; group B) suture, respectively. Antibacterial prophylaxis, painkillers, and anticoagulant were used in all patients postoperatively. Wound dressing was changed once in 3 days, and the stitches were taken out 14 days after surgery in group B. All patients received the same rehabilitation postoperatively (Figs. 1 and 2).

### 2.3 Parameters

The Knee Society score (KSS) was assessed before and 6 months after the operation. Inflammatory markers including serum levels of procalcitonin (PCT), interleukin (IL)-6, and C-reactive protein (CRP), and the erythrocyte sedimentation rate (ESR) were measured before, 1 day postsurgery, and 1 month after operation. Patient satisfaction with skin closure was evaluated using the Likert scale at the last follow-up. In addition, all surgery and postoperation hospital records were also collected.

### 2.4 Statistical analysis

The data were analyzed by the IBM SPSS 19.0 statistical software. Age, BMI, KSS, inflammation marker levels, and Likert scores were compared using independent-samples *t* test. The Chi-squared test was used to assess categorical variables like sex. *P* < .05 was considered statistically significant.

## 3 Results

The 6-month postoperative clinical (88.3 ± 5 vs 86.8 ± 4.5) and functional (74.4 ± 10.3 vs 70.9 ± 9.6) KSS were similar between the running subcuticular and intermittent closure groups. Likewise, the duration of postoperation hospitalization also did not show any significant difference between both groups (running subcuticular closure 5.7 ± 1.3 days vs 5.2 ± 1.6 days intermittent closure). However, patients that received the running subcuticular closure expressed greater satisfaction in terms of the Likert score compared to the intermittent closure group (4 ± 1.0 vs 3.6 ± 1.2; *P* < .05). The inflammation-related markers were also not significantly different between the 2 groups (Reviewer #1 point 4). For instance, PCT levels 1-day and 1-month postoperation were, respectively, 0.06 ± 0.04 μg/L and 0.06 ± 0.13 μg/L in the running subcuticular closure group, and 0.07 ± 0.06 μg/L and 0.05 ± 0.04 μg/L in the intermittent closure group. Similarly, the IL-6 levels at the above time points were 43.35 ± 34.86 pg/mL and 6.19 ± 3.83 pg/mL in the running subcuticular closure group, and 55.12 ± 38.72 pg/mL and 7.79 ± 7.68 pg/mL in the intermittent closure group. Postoperative CRP levels were, respectively, 27.00 ± 23.28 mg/L and 9.29 ± 11.48 mg/L after 1 day and 1 month in the running subcuticular closure group, and 32.96 ± 22.21 mg/L and 17.33 ± 34.08 mg/L in the intermittent closure group. Finally, the 1-day and 1-month postoperative ESR in the running subcuticular closure group were 46.53 ± 33.01 mm/h and 55.63 ± 31.07 mm/h, respectively, and 38.86 ± 31.07 mm/h and 62.52 ± 30.59 mm/h in the intermittent closure group at the same time points. In addition, no severe adverse events occurred in either group except for 1 case in the running subcuticular closure group that showed superficial infection 3 months after the operation. The patient was treated with sensitive antibiotics, debridement, and polyethylene liner replacement (Reviewer #1 point 4, Reviewer #1 point 6) (Tables 1–3).

**Table 1:**
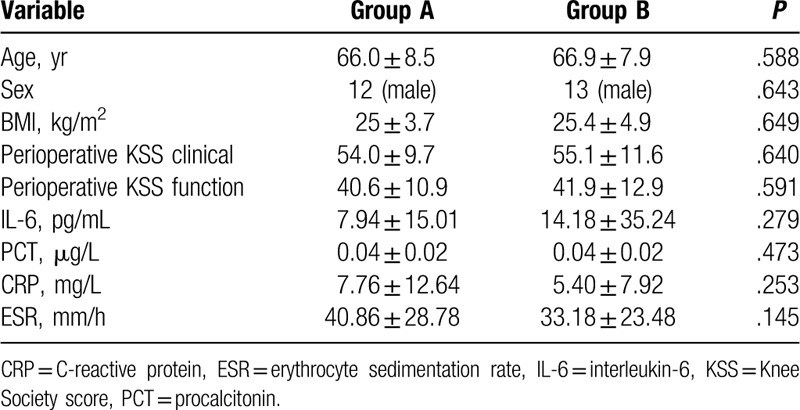
Comparison between 2 types of closures preoperative.

**Table 2:**
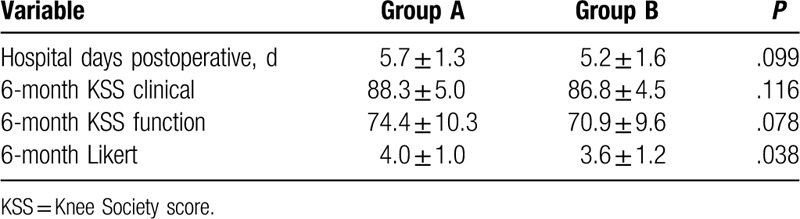
Comparison between 2 types of closures postoperative.

**Table 3:**
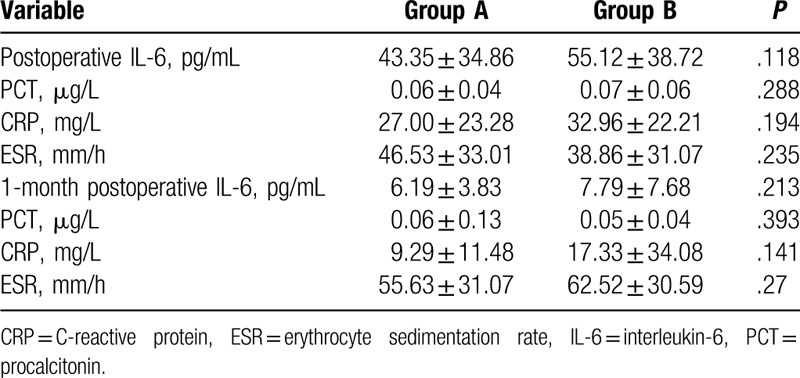
Comparison between 2 types of closures postoperative.

## 4 Discussion

Wound closure after TKA affects the outcome and risk of infection. We compared 2 common techniques of wound closure in 101 patients, and did not observe any significant differences in the 6-month follow-up KSS or inflammation markers. However, the subjective Likert score was higher for patients who underwent running subcuticular closure compared to those that received an intermittent closure.

The outcomes of TKA are pain relief and improved range of motion and function of the knee. Rehabilitation training is necessary following TKA^[10,11]^ to prevent joint contracture, deep vein thrombosis (Reviewer #1 point 7) or hypostatic pneumonia. The tension is distributed uniformly along the entire incision in running subcuticular closure, whereas that in the intermittent closure method is restricted to the elevated stitch compared to the region between consecutive stitches. This difference in tension distribution can potentially affect early pain and the range of knee motion, resulting in different outcomes. However, we found no significant differences in the outcomes between both suture techniques, indicating that the latter is not a determinant of TKA efficacy. This is consistent with previous studies that have compared various wound closure methods used in TKA and found no major differences in terms of the 6-month outcome. However, Smith et al reported a higher incidence of superficial dehiscence with barbed sutures.^[6]^

Cosmetic appearance postsurgery is an important part of patient satisfaction, and as per the Likert scale, was higher in patients that received absorbable sutures. This is likely due to the fact that the rough surface of unabsorbable silk suture can lead to aseptic inflammation of the local skin, and its uneven tension results in the formation of centipede-like scar perpendicular to the suture. In addition, removal of these stitches leaves “pinholes” that can occasionally cause a localized skin inflammation.

Periprosthetic infection is a serious complication of TKA that incurs extensive costs and poor prognosis.^[12,13]^ The running subcuticular closure results in better skin and soft-tissue perfusion close to the incision compared to intermittent closure,^[14]^ which accelerates wound healing. Furthermore, the tighter suture used in intermittent closure not only worsens perfusion but also prevents drainage of the liquefied subcutaneous fat, which may lead to subcutaneous necrosis and infection^[15]^ (Reviewer #1 point 4). We evaluated the levels of CRP, ESR, PCT, and IL-6, which are reliable makers of SSIs,^[16,17]^ and found no differences between 2 groups. This can be attributed to the imperceptible antibacterial effect of absorbable sutures, as well as the elevated IL-6, CRP, and ESR following stress response postoperation (Reviewer #1 point 5). Yoon et al conducted a meta-analysis of 18 studies including 1835 subjects, a high positive likelihood ratio were found in both IL-6 and PCT test indicated both of them may be a good rule-in tests for the diagnosis of periprosthetic joint infection^[18]^ (Reviewer #1 point 3). Lin et al recently concluded a prospective, randomized, open-label clinical trial on the risk of SSIs in patients that received triclosan-coated or plain polyglactin sutures after TKA, and detected lower levels of IL-6 with the former between 4 weeks and 3 months after operation.^[19]^

All the surgeries included in our study were performed by one surgeon, thus eliminating any possible bias due to individual variations. Furthermore, the risk of infection was assessed using objective indicators like IL-6, PCT, ESR, and CRP which eliminated observer bias. However, the retrospective nature of the study was a limitation, along with heterogeneity among the patients and the inaccuracy of the time of wound closure. Some potentially confounding variables like self-healing ability, history of using corticosteroids, or incomplete medical records were also not considered. Nevertheless, we can conclude from our findings that the running subcuticular closure results in better cosmetic appearance compared to intermittent closure, without affecting the 6-month outcome and risk of SSIs.

## 5 Conclusion

Running subcuticular closure after TKA results in a better appearance compared to intermittent closure, although neither method has an advantage in terms of function and risk of infection.

## Author contributions

Liang Chen and Min Zeng designed the study.

Jie Xie, Yihe Hu, and Min Zeng performed the surgeries.

Liang Chen, Junxiao Yang, and Min Zeng collected and analyzed the data.

Liang Chen and Min Zeng wrote the main manuscript.

All authors reviewed the manuscript.
